# Are the 2009 Institute of Medicine gestational weight gain recommendations applicable in a contemporary South-East Asian pregnancy cohort? Results of a prospective analysis

**DOI:** 10.1371/journal.pone.0316837

**Published:** 2025-01-06

**Authors:** Yong Ting Tai, Jun Kit Khoo, Quan Hziung Lim, Lee-Ling Lim, Sharmila Sunita Paramasivam, Jeyakantha Ratnasingam, Nicholas Ken Yoong Hee, Tharsini Sarvanandan, Ying Guat Ooi, Victoria Wei Fang Boey, Saravanaa Nalliah, Peng Chiong Tan, Mukhri Hamdan, Pavai Sthaneshwar, Nurshadia Samingan, Azanna Ahmad Kamar, Azriyanti Anuar Zaini, Syahrizan Samsuddin, Md Syazwan Md Amin, Nurbazlin Musa, Shubash Shander Ganapathy, Karuthan Chinna, Muhammad Yazid Jalaludin, Shireene Ratna Vethakkan

**Affiliations:** 1 Endocrine Unit, Department of Medicine, Faculty of Medicine, Universiti Malaya, Kuala Lumpur, Malaysia; 2 Ministry of Health, Putrajaya, Malaysia; 3 Department of Obstetrics & Gynaecology, University Malaya Medical Centre, Kuala Lumpur, Malaysia; 4 Department of Pathology, University Malaya Medical Centre, Kuala Lumpur, Malaysia; 5 Department of Paediatrics, Faculty of Medicine, University Malaya, Kuala Lumpur, Malaysia; 6 Institute for Public Health, National Institutes of Health, Ministry of Health Malaysia, Shah Alam, Selangor, Malaysia; 7 Faculty of Business and Management, UCSI University, Kuala Lumpur, Malaysia; Aga Khan University, PAKISTAN

## Abstract

Gestational Weight Gain (GWG) modulates pregnancy outcomes and long-term offspring metabolic health. The 2009 Institute of Medicine (IOM) GWG recommendations have largely been validated in Caucasian and mono-ethnic East Asian cohorts. Asians are at higher metabolic risk at a lower body mass index (BMI), and this has prompted the World Health Organization (WHO) to identify lower BMI cut-offs for risk evaluation amongst Asians. This prospective observational cohort study aimed to determine if 2009 IOM GWG thresholds are applicable in a contemporary multi-ethnic South-East Asian cohort. We recruited 875 mothers from an urban Malaysian tertiary clinic during screening for gestational diabetes mellitus (GDM) from 2014–2021. Data collected included measures of insulin-sensitivity, total GWG (maternal weight at delivery–self-reported pre-gravid weight), and neonatal anthropometrics (birthweight and skinfold-thickness measured with Harpenden calipers). BMI was stratified by Caucasian (overweight ≥25kg/m^2^, obese ≥30kg/m^2^) as well as Asian (overweight ≥23kg/m^2^, obese ≥27.5kg/ m^2^) cut-offs, and patients categorized by 2009 IOM GWG reference ranges. The cohort comprised 67% Malay-, 23% Chinese- and 10% Indian-descent mothers with a high prevalence of overweight/obesity (Asian cut-offs 56.9% vs Caucasian 44%). When Asian BMI cut-offs were deployed, excessive GWG incidence increased (34.1% → 40.6%) whilst inadequate GWG declined (30% → 24.8%) (p<0.05). Upon multivariate-analysis (adjusting for age, parity, race, GDM, insulin-sensitivity, baby-gender) excessive GWG categorized with Caucasian BMI cut-offs was significantly associated with increased risk of macrosomia (adjusted odds ratio (aOR) 8.65, 95% confidence interval (CI) 1.07–70.01), Neonatal-Fat-Mass (NFM) >90th centile (aOR 2.14, 95% CI 1.02–4.45) and Sum-of-Skinfold Thickness (SSFT) >90th centile (aOR 3.88, 95% CI 1.77–8.51). Excessive GWG by Asian cut-offs was also associated with increased risk of SSFT >90th centile (aOR 5.75, 95% CI 2.35–14.10). Inadequate GWG by both Caucasian and Asian BMI cut-offs was associated with Small-for-Gestational-Age (SGA) status (aOR 4.30, 95% CI 2.48–7.45 and aOR 3.66, 95% CI 2.13–6.30 respectively). In conclusion, the 2009 IOM GWG recommendations, using either Caucasian or regional Asian BMI cut-offs, are applicable in a contemporary Malay majority South-East Asian cohort in terms of predicting abnormal neonatal adiposity. Importantly, the association with neonatal adiposity is independent of increased maternal insulin resistance characteristic of Asians.

## Introduction

Inappropriate Gestational-Weight-Gain (GWG) is a modifiable risk factor not just for adverse pregnancy outcomes but also long-term maternal-child metabolic health [1, 2]. Excessive and inadequate GWG are associated with large-for-gestational-age (LGA), Caesarean delivery, gestational-diabetes-mellitus (GDM), pre-eclampsia, small-for-gestational-age (SGA) and premature delivery [1]. Long-term metabolic consequences of abnormal GWG for offspring include childhood/adult obesity and dysglycaemia [1, 2]. Additionally, abnormal GWG is also linked with post-partum weight retention, long-term cardio-metabolic risk and post-partum depression in the mother [1]. It is now well-established that ante-natal lifestyle interventions can impact on GWG, hence modifying risk of developing outcomes such as GDM/Caesarean delivery [3]. Given the developmental origins of health and disease and being cognizant of the role played by the intra-uterine milieu in determining offspring health trajectories in childhood/adulthood, it is therefore vital to establish clinically appropriate GWG thresholds to guide antenatal care [4].

The United States 2009 IOM (recently renamed the National Academy of Medicine) GWG guidelines are based on historical data derived mainly from White North American women and might not be applicable to Latino/Asian populations [1, 5]. Since their promulgation in 2009, they have been adopted by the American College of Obstetricians and Gynaecologists and widely applied globally [1]. However, it is not known if these guidelines are universally applicable. While 2009 IOM GWG recommendations have been extensively validated in Western countries, there is a dearth of data on the impact of GWG in Asian antenatal cohorts, especially in the Indian subcontinent and South-East Asia [1, 2]. The few publications and meta-analyses exploring use of 2009 IOM recommendations amongst Asian women and finding them to be applicable using both Caucasian and Asian BMI cut-offs, have been mainly derived from mono-ethnic East Asian countries (China, Taiwan, Korea, Japan) which are known to have unusually high prevalence rates of underweight, hence skewing maternal BMI towards the lower range of the spectrum [2, 3, 6, 7]. It is not known if the conclusions drawn from these analyses can be extrapolated to all Asian ethnicities, or similar Asian ethnicities who have migrated to countries where environmental obesogenicity, health-care systems, cultural practices that impact on diet, belief systems and other socio-economic determinants of health differ [1, 3]. Therefore the application of IOM guidelines, developed in North America or validated amongst migrant Asians living in the West, requires further scientific justification with evidence derived from regional data before deployment in individual Asian countries/regions.

Asians comprise 60% of the global population and have a higher metabolic risk profile at a lower BMI when compared with Caucasians [8, 9]. These same differences in metabolic phenotype amongst non-gravid Asians may also translate into increased metabolic risk during pregnancy with concomitant suboptimal pregnancy outcomes. Adult non-gravid Asians have shorter stature, higher fat mass/increased visceral adiposity, increased insulin resistance (secondary to higher circulating free fatty acids/inflammation) and greater beta cell secretory dysfunction at a lower BMI [1, 8–10]. Hence the WHO recommends lower BMI risk thresholds in Asians, compared with Caucasians [9]. It is unclear if these lower BMI risk thresholds should also be applied in pregnancy. Indeed, historical epidemiological data, once again derived from predominantly East Asian women, reveal the lowest maternal obesity/GWG rates amongst Asians globally, along with the highest prevalence of underweight [11].

Malaysia is a middle-income developing South-East Asian country with an ethnically diverse population comprised of Malay, Chinese and Indian subgroups [12]. The native Malay population which is the majority ethnic group in both Malaysia and Indonesia (the 4^th^ most populous nation) has been particularly under-studied. The Chinese and Indians are descendants of migrant populations who settled Malaysia over a period from the 19^th^—early 20^th^ century. Given the substantial role played by GWG as a determinant of maternal-foetal health, it is important to delineate region-specific thresholds in a cohort residing in South-East Asia where the biopsychosocial milieu may differ from the West/home-countries of migrant groups. The few existing studies conducted in Malaysia, Singapore, Indonesia and Thailand have conflicting results, with 1 study finding Western BMI cut-offs to be applicable for both reduced and increased neonatal adiposity, 1 for only increased adiposity and 2 for only reduced adiposity, hence underscoring the need for more data to clarify this thorny issue [13–16]. Additionally, in the last two decades, many Asian countries including Malaysia have undergone socio-economic/dietary transitions secondary to globalization resulting in increasing maternal obesity–hence it is also necessary to establish that the 2009 guidelines based on a historical cohort are applicable in modern times. Prior existing South-East Asian research were conducted a decade or more earlier, necessitating reassessment of the changes that have occurred in the intervening years since publication [13–16].

Malaysia has the dubious distinction of being the most obese nation in South-East Asia and even Asia, with a rise in the prevalence of overweight/obesity from 44.5% in 2015 to 54.4% in 2023 [12, 17]. The national increase in obesity prevalence is mirrored by increased maternal overweight/obesity in the antenatal cohort, with 2/3rds of Malaysian mothers at booking having a BMI >23 kg/m^2^ [18]. Examples of recent changes in food culture that have accelerated this rise in maternal obesity include the rise in food-delivery services and fast-food outlets, such that 40% of Malaysians now consume food outside the home daily, 20% consume fast-food at least once a week and 95% admitted to inadequate fruit/vegetable intake [12]. The carbohydrate staple anchoring the Malaysian diet has traditionally been rice; however modernization/ Westernization has witnessed a ‘nutrition transition’ ie an increase in wheat, sugar, animal protein and processed food consumption with a concomitant fall in rice/plant-based protein consumption [19]. Aside from dietary and occupational changes, the modern obesogenic built environment in Malaysian cities and suburbs has also contributed to a rise in BMI [12]. A global study that included data from Malaysia, has revealed that high traffic density and community disorder are positively associated with obesity whilst bike lanes and pedestrian safety (leading to increased ‘walkability’) are inversely related to obesity [20].

Therefore, we designed this prospective observational cohort study aiming to explore the validity of applying 2009 IOM GWG guidelines (with and without modification using Asian BMI cut-offs), in a multi-ethnic cohort of Malaysian mothers. The approach of transposing Asian BMI cut-offs on to IOM GWG ranges was based on similar methods used by 2 East Asian studies (Wie et al. & Guan et al.) and a review by Goldstein et al. [3, 6, 7]. We hypothesized that when Malaysian mothers are categorised by either Caucasian or Asian BMI cut-offs using the same GWG reference ranges proposed by the 2009 IOM guidelines, there will be a significant association of inappropriate GWG with abnormal neonatal anthropometrics. It is hoped that our data derived from a much under-studied geographical region of South-East Asia and the hitherto neglected Malay ethnic subgroup will contribute to global healthcare.

## Methods

### Study design

In this single-centre prospective observational cohort study, we recruited 875 women at the point of their screening oral glucose tolerance test (OGTT) for GDM from an antenatal clinic (ANC) of a tertiary hospital in Kuala Lumpur, Malaysia from 12 February 2014 to 11 January 2021. All women provided written informed consent. The study was conducted in accordance with the Declaration of Helsinki and approved by the local ethics committee. Participants were recruited by convenience sampling. We enrolled only Malaysian-born women (Malay, Chinese or Indian descent), ≥18 years old with a singleton pregnancy (14–32 weeks gestation) with either normal glucose tolerance (NGT) or GDM, who received antenatal care/delivered at the aforementioned centre. Women with pre-gestational diabetes/overt diabetes diagnosed in pregnancy were excluded. (Refer to Table 1 for details).

**Table 1:** Inclusion and exclusion criteria.

Inclusion Criteria • ≥18 years old • Singleton pregnancy • NGT or GDM (FPG≥ 5.1 mmol/l and/or 2hr glucose ≥7.8 mmol/l)* • Malaysian-born Malay, Chinese or Indian woman
Exclusion Criteria • Pre-gestational diabetes • Overt diabetes diagnosed in pregnancy (fasting plasma glucose [FPG] ≥ 7.0 mmol/L, HbA1c ≥ 6.5% and/or random plasma glucose [RPG] ≥ 11.1 mmol/L)* • Assisted conception • Multiple pregnancy • Active chronic systemic disease • Hypo-/hyperthyroidism • Cushing’s syndrome (or exogenous steroids for treatment of diseases such as poorly controlled asthma, systemic lupus etc) • Infection (HIV, Hepatitis B/C, tuberculosis) • Pregnancies with foetal anomalies • Pregnancies with imminent/preterm delivery due to maternal diseases (besides GDM/pregnancy-induced hypertension)

HIV, Human Immunodeficiency Virus

*screening for GDM conducted with 2 time-point 2-hour 75-gram OGTT as per 2017 Malaysian Clinical Practice Guidelines [21]

At recruitment, demographic, anthropometric (height/weight) and clinical data were obtained during an interview/examination using a structured questionnaire. BMI was calculated based on height measured and self-reported pre-gravid weight. Women were then stratified by Caucasian (overweight ≥25 kg/m^2^, obese ≥30 kg/m^2^) and Asian (overweight ≥23 kg/m^2^, obese ≥27.5 kg/m^2^) BMI cut-offs. Additional blood was drawn for fasting insulin during the 75-gram OGTT. During the second visit at 36 weeks gestation, maternal weight was measured once again. In GDM mothers, HbA1c was evaluated at 36 weeks.

At the 3rd encounter, post-delivery, newborn anthropometric-measurements, including birth weight (BW) and skinfold-thickness, were conducted by the two same trained research assistants, according to standardized procedures within 24 hours of birth. Neonatal-fat-mass (NFM) and sum-of-skinfold-thickness (SSFT) were calculated using validated formulae (S1 Appendix). Information regarding total GWG, maternal weight at delivery, gestation at delivery, mode of delivery and neonatal outcomes were collected based on interview/case records.

Details of screening, diagnosis, and standard of care for GDM at our centre are given in S1 Appendix. A detailed account of biochemical analyses and neonatal measurement methods is found in S1 Appendix.

### Definitions and calculations

#### Maternal parameters and measures

**Pre-pregnancy BMI:** self-reported weight in kg prior to pregnancy divided by measured height in meter squared.

Pre-pregnancy BMI classification:

**Table:** 

BMI Categories [22]	Asian/Malaysian	Caucasian
Underweight	< 18.5 kg/m^2^	< 18.5 kg/m^2^
Normal	18.5–22.9 kg/m^2^	18.5–24.9 kg/m^2^
Overweight	23–27.4 kg/m^2^	25–29.9 kg/m^2^
Obese	≥ 27.5 kg/m^2^	≥ 30 kg/m^2^

*HOMA2-%S*. HOMA2-%S (Updated Homeostasis Model for Assessment of Insulin Sensitivity) was computed using the HOMA2 calculator available on the website of Radcliffe Department of Medicine, Medical Sciences Division, University of Oxford [23].

**Total Gestational weight gain (GWG)** = Maternal final weight before delivery—Pre-pregnancy self-reported weight

*GWG categories*. Mothers were categorized according to the original 2009 IOM GWG recommendations and definitions of inappropriate GWG using Caucasian pre-pregnancy BMI cut-offs as tabulated below.

**Table:** 

Pre-pregnancy BMI (Caucasian)	Inadequate GWG (kg)	Appropriate Gestational Weight Gain (kg)	Excessive GWG (kg)
Underweight (<18.5 kg/m^2^)	<12.5	12.5–18	>18
Normal weight (18.5–24.9 kg/m^2^)	<11.5	11.5–16	>16
Overweight (25.0–29.9 kg/m^2^)	<7	7–11.5	>11.5
Obese (≥30.0 kg/m^2^)	<5	5–9	>9

For comparison, GWG was also categorized using a modification of the 2009 IOM recommendations that transposed Asian pre-pregnancy BMI categories on to the original GWG reference ranges as tabulated below.

**Table:** 

Pre-pregnancy BMI (Asian)	Inadequate GWG (kg)	Appropriate Gestational Weight Gain (kg)	Excessive GWG (kg)
Underweight (<18.5 kg/m^2^)	<12.5	12.5–18	>18
Normal weight (18.5–22.9 kg/m^2^)	<11.5	11.5–16	>16
Overweight (23.0–27.4 kg/m^2^)	<7	7–11.5	>11.5
Obese (≥27.5 kg/m^2^)	<5	5–9	>9

Neonatal outcomes and measures.

**Premature delivery**: delivery at less than 37 completed weeks of gestation

**Macrosomia**: BW ≥ 4000g

**Low birth weight**: BW < 2500g

**BW centile**: determined using gestational age- and sex-adjusted BW charts based on Fenton 2013 growth charts.

- Small for gestational age (SGA): BW < 10^th^ centile
- Average for gestational age (AGA): BW between 10th– 90^th^ centile
- Large for gestational age (LGA): BW > 90^th^ centile

**Sum-of-Skinfold-Thickness (SSFT) & Neonatal-fat-mass (NFM)**: calculated based on the formula derived from Catalano et al. [24] (S1 Appendix).

### Outcomes

The pre-specified primary outcomes were incidence of LGA, Macrosomia, SGA, low BW and BW/NFM/SSFT below the calculated 10^th^ centile and above the calculated 90^th^ centile for this cohort. Incidence of Caesarean delivery was a secondary outcome.

### Statistical analysis

The sample size calculation was based on a prevalence rate of 47% excessive GWG, with RR of 1.85 of LGA associated with excessive GWG, derived from the meta-analysis data of Goldstein et al. with an alpha of 5% and power of 80% [3]. The calculated sample size is therefore 886.

Data are presented as mean ± standard deviation (SD), median (interquartile range [IQR]) or frequency (percentage), as appropriate. Comparison of means for more than 2 groups was done using one-way Analysis of Variance (ANOVA) with post-hoc Scheffe’s or Tukey’s corrections for significant difference. Categorical data are expressed as frequency and percentage and analysed using Chi-Square test. Customized thresholds in our sample population for BW, NFM and SSFT > 90^th^ centile were calculated. The strength of association between inappropriate GWG (categorized using Caucasian and Asian pre-pregnancy BMI cut-offs) with neonatal outcomes was evaluated. Bivariate correlations were used to determine the relationship between maternal parameters and neonatal outcomes. The logistic regression model was performed to measure the potential risk factors by calculating the odds ratio (OR) and 95% confidence interval (CI). Univariable analysis of predictors was conducted, and outcomes with p < 0.25 were considered in the multivariable analyses. The final model included age, ethnicity, parity, pre-pregnancy BMI, GWG, GDM status, HOMA2-%S and baby gender as the main variables. The final model was tested for multicollinearity and correlation between significant variables. The model was also tested for fitness of model, Hosmer-Lemeshow > 0.05. Analyses were performed with IBM Statistical Package for Social Sciences (SPSS) version 25 with two-tailed p < 0.05 considered statistically significant.

## Results

**Fig 1:**
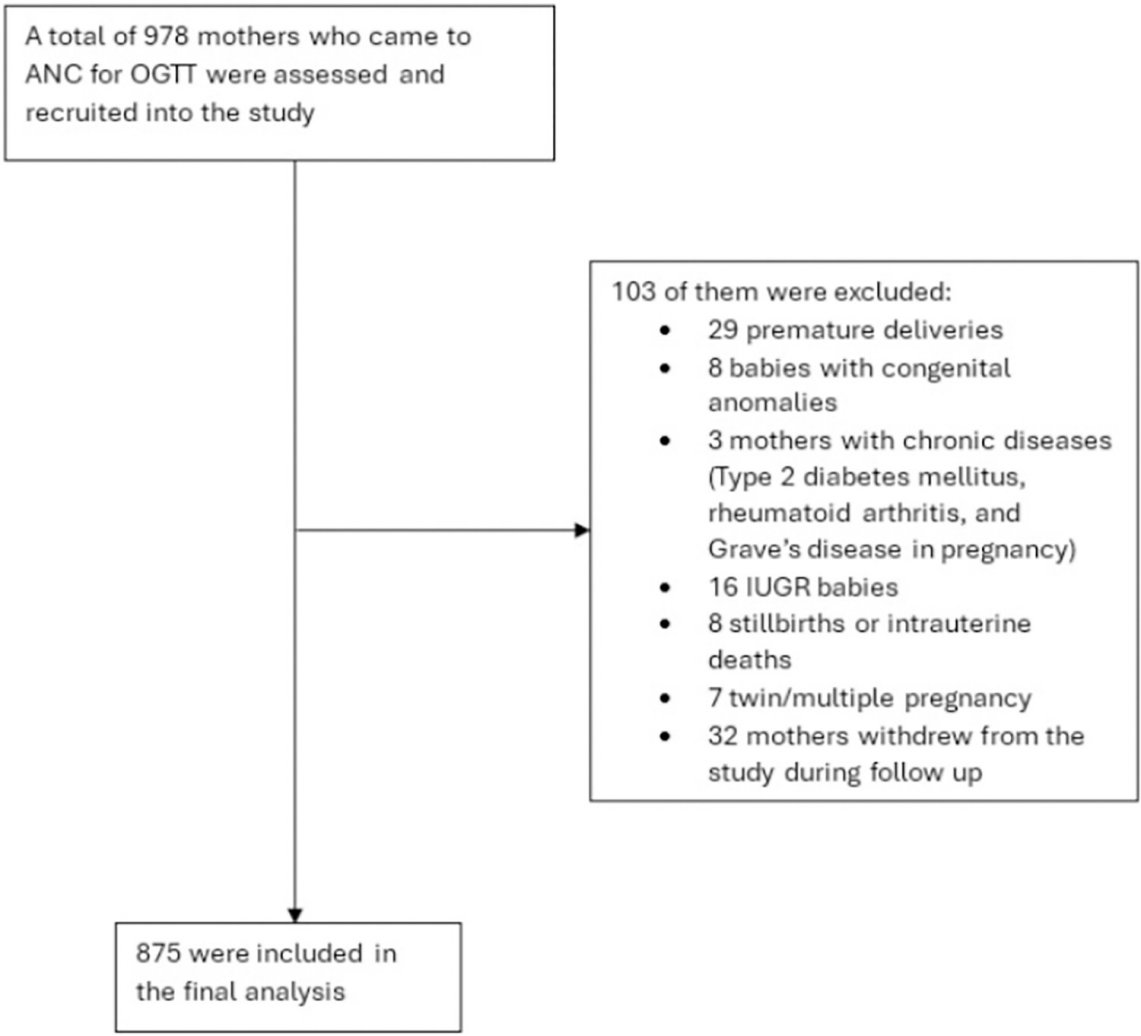
Flow diagram for the study cohort.

A total of 978 mothers were assessed and recruited into the study, 103 were excluded (71 did not fulfil the inclusion/exclusion criteria, 32 withdrew from the study), as shown in Fig 1. Hence only 875 were included in the final analysis. Mean age was 31.6 ± 4.6 years. Majority of the cohort were of Malay ethnicity (66.9%), followed by mothers of Chinese (23.3%) and Indian (9.8%) ethnicity. 29.7% were diagnosed with GDM, while only 7.3% developed PIH. 7% were underweight. Using Caucasian BMI cut-offs, 49% were normal-weight, and 44% overweight/obese. When mothers were re-categorised using Asian BMI cut-offs, prevalence of overweight/obesity increased to 56.9%. A negligible number of women reported a history of smoking or alcohol use prior to conception (< 2%) and the majority had at least secondary education status (94.4%) (Table 2).

**Table 2:** Maternal demographic, anthropometric and clinical characteristics.

Demographic, anthropometric, and clinical characteristics	N = 875 (%)
Maternal age (years), Mean ± SD	31.6 ± 4.6
Race, N (%)	
Malay	585 (66.9)
Chinese	204 (23.3)
Indian	86 (9.8)
Pre-pregnancy BMI (kg/m^2^), Mean ± SD	25.0 ± 5.4
Asian Classification, N (%)	
<18.5	61 (7.0)
18.5–22.9	316 (36.1)
23.0–27.4	237 (27.1)
≥27.5	261 (29.8)
Caucasian Classification, N (%)	
<18.5	61 (7.0)
18.5–24.9	429 (49.0)
25.0–29.9	224 (25.6)
≥30.0	161 (18.4)
Parity, N (%)	
Primigravida	316 (36.1)
Multiparous	559 (63.9)
Maternal GDM, N (%)	
Yes	260 (29.7)
No	615 (70.3)
Maternal PIH, N (%)	
Yes	64 (7.3)
No	811 (92.7)
History of Smoking, N (%)	
Yes	6 (0.7)
No	869 (99.3)
History of Alcohol, N (%)	
Yes	13 (1.5)
No	862 (98.5)
Education Level, N (%)	
Primary	5 (0.6)
Secondary	201 (23.0)
Tertiary	661 (75.5)
Post-graduate	8 (0.9)

BMI, body mass index; PIH, pregnancy-induced hypertension. Continuous data expressed as mean ± SD. Categorical data expressed as percentages.

### Adequacy of GWG

Mean total GWG was 12.2 ± 5.0kg. When the 2009 IOM guidelines were deployed using Caucasian BMI cut-offs, incidence of adequate total GWG was 35.9%, inadequate 30% and excessive 34.1%. However, when mothers were recategorized using Asian BMI cut-offs, excessive GWG incidence increased (34.1% → 40.6%) whilst inadequate GWG declined (30% → 24.8%) (p<0.05) (Table 3).

**Table 3:** Adequacy of gestational weight gain by 2009 IOM guidelines using Caucasian & Asian BMI cut-offs.

	Inadequate N (%)	Adequate N (%)	Excessive N (%)	p-value
GWG by IOM recommendations (Asian BMI cut-offs)	217 (24.8)	303 (34.6)	355 (40.6)	<0.001
GWG by IOM recommendations (Caucasian BMI cut-offs)	263 (30.0)	314 (35.9)	298 (34.1)	
Rate of GWG in 2^nd^/3^rd^ trimester by IOM recommendations (Asian BMI cut-offs)	200 (23.0)	249 (28.6)	422 (48.4)	<0.001
Rate of GWG in 2^nd^/3^rd^ trimester by IOM recommendations (Caucasian BMI cut-offs)	236 (27.1)	273 (31.3)	362 (41.6)	

Within group differences in rates of inadequate, adequate, and excessive GWG were analysed using McNemar’s test.

### Neonatal characteristics

Mean BW was 3081.0 ± 379.2g. Only 11 cases (1.3%) of macrosomia were observed. 2.2% of neonates were categorized as LGA and 11.2% as SGA. One-third were delivered via lower segment Caesarean section (LSCS). The 90^th^ centile of the cohort for BW, NFM and SSFT were 3590g, 617.2g and 21mm respectively (Table 4).

**Table 4:** Neonatal characteristics and caesarean section incidence.

Neonatal characteristics and outcomes	N	Mean ± SD / N (%)
Gestation at delivery (weeks)	875	38.3 ± 1.1
Birthweight (g)	875	3081.0 ± 379.2
Fat mass (g)	421	434.2 ± 143.8
Sum of skinfold thickness (mm)	418	16.1 ± 3.6
Baby gender	875	
Male		447 (51.1)
Female		428 (48.9)
Baby size	875	
SGA		98 (11.2)
AGA		758 (86.6)
LGA		19 (2.2)
Macrosomia	875	
BW <2.5kg		48 (5.5)
BW 2.5–3.9kg		816 (93.3)
BW ≥4kg		11 (1.3)
Birth weight	875	
BW <10^th^ centile (<2628g)		87 (10.0)
BW 10 – 90^th^ centile (2628g – 3590g)		702 (80.2)
BW >90^th^ centile (>3590g)		86 (9.8)
Fat mass (g)	421	
NFM <10^th^ centile (<264.3g)		42 (10.0)
NFM 10 – 90^th^ centile (264.3g – 617.2g)		337 (80.0)
NFM >90^th^ centile (>617.2g)		42 (10.0)
Sum of skinfold thickness (mm)	418	
SSFT <10^th^ centile (<11.5mm)		40 (9.6)
SSFT 10 – 90^th^ centile (11.5–21.0mm)		338 (80.8)
SSFT >90^th^ centile (>21.0mm)		40 (9.6)
LSCS, N (%)	875	
Yes		301 (34.4)
No		574 (65.6)

Continuous data expressed as mean ± SD. Categorical data expressed as percentages. The 10^th^ and 90^th^ centile of birth weight, neonatal fat mass, neonatal sum of skinfold thickness for this cohort were determined using SPSS.

### Correlation analyses of GWG with neonatal outcomes

#### Univariate analysis

There was a significant gradient of increased risk across gestational weight gain categories for abnormal neonatal anthropometrics with use of both Asian and Caucasian BMI cut-offs (S1 and S2 Tables). Upon univariate analysis (Tables 5 and 6), significant associations of increased neonatal anthropometrics were higher fasting glucose at screening OGTT, low insulin sensitivity, higher fasting insulin, excessive total GWG by 2009 IOM guidelines (categorized by either Caucasian or Asian BMI cut-offs), increased total GWG and higher pre-pregnancy BMI. Factors associated with reduced neonatal adiposity included inadequate total GWG by 2009 IOM guidelines (categorized by either Caucasian or Asian BMI cut-offs), reduced total GWG, and lower pre-pregnancy BMI. GDM status was not associated with neonatal adiposity in this cohort of well-managed mothers with mean 3^rd^ trimester HbA1c of 5.4 ± 0.5%, 34.6% and 18.5% of whom were on metformin and insulin therapy respectively (S4 Table). Neither was the presence of maternal PIH significantly associated with neonatal adiposity. There was no significant difference in crude LSCS rate between GWG categories (S1 and S2 Tables).

**Table 5:** Simple logistic regression analysis for associations of increased neonatal anthropometrics.

Predictive factors	OR (95% CI)				
	BW >90^th^ centile	LGA	Macrosomia	NFM >90^th^ centile	SSFT >90^th^ centile
Age	1.02 (0.98–1.07)	1.03 (0.94–1.14)	1.00 (0.88–1.14)	1.05 (0.98–1.13)	1.05 (0.98–1.13)
Ethnicity: Malay (reference)					
Chinese	0.78 (0.45–1.37)	0.61 (0.17–2.14)	0.32 (0.04–2.50)	0.41 (0.16–1.10)	0.90 (0.41–2.00)
Indian	0.88 (0.41–1.91)	0.97 (0.22–4.35)	0.75 (0.09–6.02)	1.85 (0.79–4.37)	1.21 (0.44–3.33)
Pre-pregnancy BMI	1.06 (1.02–1.10)	1.10 (1.03–1.19)	1.10 (1.01–1.21)	1.09 (1.03–1.14)	1.05 (0.99–1.11)
Parity	1.20 (1.03–1.40)	1.26 (0.94–1.69)	1.37 (0.96–1.97)	1.19 (0.94–1.51)	1.07 (0.83–1.37)
GDM	1.03 (0.63–1.67)	2.17 (0.87–5.40)	0.89 (0.23–3.36)	1.47 (0.77–2.81)	0.60 (0.29–1.27)
PIH	1.56 (0.74–3.28)	2.44 (0.69–8.620)	1.27 (0.16–10.09)	1.49 (0.49–4.51)	1.10 (0.32–3.80)
GWG according to IOM recommendations (Asian BMI)					
Inadequate	0.46 (0.22–0.96)	0.28 (0.03–2.38)	*	0.08 (0.01–0.63)	0.16 (0.19–1.32)
Adequate (reference)					
Excessive	1.44 (0.88–2.35)	2.26 (0.80–6.41)	8.73 (1.11–68.55)	1.96 (0.97–3.96)	4.57 (1.95–10.73)
GWG according to IOM recommendations (Caucasian BMI)					
Inadequate	0.45 (0.23–0.91)	0.24 (0.03–2.04)	*	0.14 (0.03–0.62)	0.18 (0.04–0.83)
Adequate (reference)					
Excessive	1.67 (1.02–2.74)	2.87 (1.01–8.14)	11.05 (1.41–86.88)	2.39 (1.19–4.80)	3.27 (1.55–6.89)
Rate of GWG in 2^nd^ and 3^rd^ trimester according to IOM recommendations (Asian BMI)					
Inadequate	0.47 (0.22–1.01)	*	*	0.07 (0.01–0.56)	0.36 (0.07–1.79)
Adequate (reference)					
Excessive	1.23 (0.74–2.00)	0.81 (0.32–2.03)	6.02 (0.77–47.31)	1.17 (0.60–2.28)	3.78 (1.61–8.86)
Rate of GWG in 2^nd^ and 3^rd^ trimester according to IOM recommendations (Caucasian BMI)					
Inadequate	0.47 (0.23–0.98)	0.16 (0.02–1.35)	*	0.06 (0.01–0.46)	0.18 (0.04–0.81)
Adequate (reference)					
Excessive	1.52 (0.92–2.51)	1.21 (0.46–3.17)	3.51 (0.75–16.39)	1.32 (0.68–2.57)	2.12 (1.02–4.38)
Metabolic Parameters at baseline					
Fasting glucose	1.46 (1.01–2.10)	1.20 (1.06–3.77)	1.84 (0.79–4.30)	1.94 (1.22–3.08)	1.01 (0.58–1.78)
Ln Fasting Insulin	1.63 (1.15–2.32)	2.22 (1.23–3.99)	2.76 (1.40–5.45)	2.03 (1.27–3.24)	1.15 (0.69–1.91)
Ln HOMA-2%S	0.50 (0.33–0.76)	0.31 (0.14–0.69)	0.19 (0.07–0.54)	0.43 (0.25–0.74)	0.82 (0.46–1.45)
Baby gender					
Male (reference)					
Female	0.66 (0.42–1.03)	-	0.87 (0.26–2.87)	0.85 (0.45–1.61)	0.67 (0.35–1.30)

*no event in the inadequate weight gain group. Skewed data (Fasting Insulin and HOMA-2%S) are natural log-transformed and are presented with the prefix ‘Ln’. OR, odds ratio; CI confidence interval. The 90th centile of BW, NFM and SSFT were 3590g, 617.15g and 21mm respectively (determined using SPSS).

**Table 6:** Simple logistic regression analysis for associations of reduced neonatal anthropometrics.

Predictive factors	OR (95% CI)				
	BW <10^th^ centile	SGA	BW <2.5kg	NFM <10^th^ centile	SSFT <10^th^ centile
Age	0.97 (0.93–1.02)	0.93 (0.89–0.98)	0.98 (0.92–1.05)	0.92 (0.85–0.99)	0.99 (0.92–1.07)
Ethnicity: Malay (reference)					
Chinese	0.80 (0.45–1.40)	0.71 (0.41–1.23)	0.66 (0.30–1.45)	1.31 (0.63–2.73)	2.14 (1.05–4.36)
Indian	1.15 (0.57–2.35)	0.97 (0.48–1.96)	1.22 (0.50–3.00)	1.63 (0.62–4.23)	1.61 (0.57–4.54)
Pre-pregnancy BMI	0.94 (0.89–0.98)	0.90 (0.86–0.95)	0.89 (0.84–0.96)	0.95 (0.90–1.01)	0.97 (0.91–1.03)
Parity	0.73 (0.59–0.90)	0.68 (0.55–0.84)	0.75 (0.57–0.98)	0.72 (0.53–0.97)	0.88 (0.67–1.16)
GDM	1.21 (0.75–1.93)	0.74 (0.46–1.21)	1.75 (0.97–3.16)	1.32 (0.69–2.53)	2.02 (1.05–3.90)
PIH	0.93 (0.39–2.23)	0.81 (0.34–1.93)	0.84 (0.25–2.77)	0.65 (0.15–2.84)	0.68 (0.16–2.99)
GWG according to IOM recommendations (Asian BMI)					
Inadequate	1.75 (1.04–2.92)	3.19 (1.91–5.32)	2.23 (1.16–4.31)	3.23 (1.48–7.08)	2.93 (1.28–6.70)
Adequate (reference)					
Excessive	0.52 (0.29–0.94)	0.70 (0.39–1.27)	0.41 (0.17–0.98)	0.65 (0.25–1.70)	1.18 (0.46–3.01)
GWG according to IOM recommendations (Caucasian BMI)					
Inadequate	2.06 (1.24–3.41)	3.76 (2.23–6.34)	2.14 (1.13–4.06)	2.46 (1.18–5.11)	1.73 (0.83–3.63)
Adequate (reference)					
Excessive	0.55 (0.29–1.05)	0.87 (0.46–1.65)	0.32 (0.12–0.89)	0.45 (0.16–1.32)	0.59 (0.23–1.53)
Rate of GWG in 2^nd^ and 3^rd^ trimester according to IOM recommendations (Asian BMI)					
Inadequate	1.34 (0.79–2.28)	1.48 (0.90–2.44)	1.20 (0.61–2.35)	2.13 (1.00–4.55)	2.40 (1.08–5.33)
Adequate (reference)					
Excessive	0.42 (0.24–0.74)	0.37 (0.21–0.64)	0.32 (0.15–0.69)	0.48 (0.20–1.16)	0.73 (0.31–1.73)
Rate of GWG in 2^nd^ and 3^rd^ trimester according to IOM recommendations (Caucasian BMI)					
Inadequate	1.91 (1.13–6.25)	2.19 (1.33–3.60)	1.68 (0.86–3.28)	2.07 (0.99–4.33)	1.81 (0.85–3.83)
Adequate (reference)					
Excessive	0.63 (0.35–1.13)	0.53 (0.29–0.95)	0.46 (0.21–1.04)	0.46 (0.18–1.19)	0.52 (0.21–1.30)
Metabolic Parameters at baseline					
Fasting glucose	0.86 (0.56–1.31)	0.51 (0.32–0.80)	1.04 (0.61–1.77)	0.77 (0.42–1.41)	0.62 (0.32–1.21)
Ln Fasting Insulin	0.89 (0.60–1.31)	0.61 (0.42–0.90)	0.81 (0.49–1.36)	1.31 (0.81–2.14)	0.61 (0.35–1.07)
Ln HOMA-2%S	1.22 (0.81–1.85)	1.75 (1.16–2.63)	1.40 (0.81–2.43)	0.78 (0.45–1.36)	2.11 (1.09–4.06)
Baby gender					
Male (reference)					
Female	1.81 (1.15–2.86)	-	1.80 (0.99–3.27)	2.03 (1.04–3.98)	1.05 (0.55–2.01)

The 10^th^ centile of BW, NFM and SSFT were 2628g, 264.26g and 11.5mm respectively (determined using SPSS).

#### Multivariate analysis

Upon multivariate analysis (adjusting for age, parity, race, GDM, insulin sensitivity, gestational age, baby gender), excessive GWG categorized by Caucasian BMI cut-offs was associated with increased risk of macrosomia (adjusted OR 8.65, 95% CI 1.07–70.01), NFM >90^th^ centile (adjusted OR 2.14, 95% CI 1.02–4.45) and SSFT >90^th^ centile (adjusted OR 3.88, 95% CI 1.77–8.51). Excessive GWG by Asian cut-offs was associated with increased risk of SSFT >90^th^ centile (adjusted OR 5.75, 95% CI 2.35–14.10). Conversely inadequate GWG also reduced the risk of fetal over-nutrition (Table 7). After adjusting for confounders, inadequate GWG was associated with SGA status by both Caucasian (adjusted OR 4.30, 95% CI 2.48–7.45) and Asian BMI cut-offs (adjusted OR 3.66, 95% CI 2.13–6.30). Inadequate GWG by both Caucasian and Asian BMI cut-offs were strongly associated with several other measures of reduced adiposity as well, ie BW < 2.5 kg, BW < 90^th^ centile, NFM < 90^th^ centile. Conversely excessive GWG also reduced the risk of reduced neonatal adiposity (Table 7). Neither inadequate nor excessive GWG was significantly associated with the crude LSCS rate upon multivariate analysis.

**Table 7:** Multiple logistic regression analysis for associations of increased and reduced neonatal anthropometrics.

Neonatal outcomes	Adjusted OR (95% CI)			
	Inadequate GWG (Asian BMI cut-offs)*	Excessive GWG (Asian BMI cut-offs)*	Inadequate GWG (Caucasian BMI cut-offs)*	Excessive GWG (Caucasian BMI cut-offs)*
BW <10th centile^¥^	1.95 (1.11–3.43)	0.53 (0.28–0.99)	2.36 (1.36–4.11)	0.55 (0.28–1.10)
SGA	3.66 (2.13–6.30)	0.70 (0.37–1.29)	4.30 (2.48–7.45)	0.79 (0.40–1.55)
BW <2.5kg	2.25 (1.12–4.50)	0.40 (0.16–0.96)	2.06 (1.05–4.03)	0.28 (0.10–0.79)
NFM <10th centile^¥^	4.19 (1.80–9.78)	0.53 (0.19–1.44)	3.04 (1.37–6.75)	0.36 (0.12–1.10)
SSFT <10th centile^¥^	2.00 (0.82–4.85)	0.94 (0.35–2.52)	1.32 (0.58–3.00)	0.64 (0.23–1.78)
BW >90th centile^¥^	0.50 (0.23–1.08)	1.13 (0.66–1.92)	0.48 (0.23–1.00)	1.50 (0.87–2.58)
LGA	0.27 (0.03–2.38)	1.94 (0.66–5.68)	0.21 (0.02–1.91)	2.58 (0.88–7.58)
Macrosomia	€	6.17 (0.76–49.71)	€	8.65 (1.07–70.01)
NFM >90th centile^¥^	0.09 (0.01–0.69)	1.80 (0.86–3.81)	0.14 (0.03–0.63)	2.14 (1.02–4.45)
SSFT>90th centile^¥^	0.15 (0.02–1.29)	5.75 (2.35–14.10)	0.17 (0.04–0.77)	3.88 (1.77–8.51)

*Adjusted for age, parity, race, GDM, Ln HOMA2%S and baby gender, ¥Adjusted for gestational age at delivery. € no event in the inadequate weight gain group. The 10^th^ centile of BW, NFM and SSFT were 2628g, 264.26g and 11.5mm respectively (determined using SPSS).

## Discussion

We observed an alarmingly high prevalence of pre-pregnancy overweight/obesity (56.9% Asian criteria, 44% Caucasian criteria) with a low prevalence of underweight (7%), in this urban multi-ethnic contemporary Malaysian cohort of 875 singleton pregnancies. Using Asian BMI cut-offs, 40.6% of mothers had excessive GWG. When Caucasian BMI cut-offs were employed, incidence rates of excessive GWG were significantly lowered while rates of inadequate GWG increased. Despite the high prevalence of maternal adiposity, there was a low incidence of increased neonatal adiposity (1.3% macrosomia [BW > 4kg], 2.2% LGA) but a higher rate of SGA (11.2%). Inappropriate GWG according to 2009 IOM recommendations was associated with a gradient of risk for both reduced and increased neonatal adiposity regardless of whether Caucasian or Asian BMI cut-offs were applied. Our study found that after comprehensively adjusting for confounders (age, ethnicity, parity, pre-pregnancy BMI, GDM status, gestation at delivery, insulin resistance), inadequate and excessive GWG, as originally defined by the 2009 IOM guidelines using Caucasian BMI cut-offs, were independently associated with risk of reduced (SGA, low birth weight < 2.5 kg) and increased (macrosomia, NFM > 90^th^ centile and SSFT > 90^th^ centile) neonatal anthropometrics respectively. When 2009 IOM guidelines were adapted by transposing Asian BMI cut-offs for obesity on to the same GWG reference-ranges, excessive GWG remained associated with increased risk of neonatal adiposity (SSFT > 90^th^ centile) and all measures of reduced adiposity (SGA, low BW, SSFT < 90^th^ centile). Overall, our findings indicate that the 2009 IOM recommendations, using both Caucasian and Asian BMI cut-offs, are applicable in our multi-ethnic Asian cohort, in terms of predicting adverse pregnancy outcomes related to neonatal adiposity. At our present sample size however, Asian BMI cut-offs are strongly associated with reduced neonatal adiposity in those with inadequate GWG, with a less robust association between increased GWG and macrosomia. This may be secondary to the low macrosomia event rate in this well managed urban tertiary care cohort. Nevertheless, the significant correlation of excessive GWG with increased SSFT we observed is highly significant clinically, given the established phenomenon of the ‘thin-fat’ Asian baby and evidence that fat mass at birth is a more discriminatory indicator of future cardio-metabolic risk in childhood than crude birth weight [25–27]. Indeed, amongst Hong Kong mothers enrolled in the HAPO study, a model of customized optimal GWG ranges derived from neonatal-fat-mass outcomes was more discriminative than IOM guidelines or models based on BW in predicting childhood BMI, waist circumference, beta cell function, insulin sensitivity and blood pressure at age 7 years [26]. This is because BW-based parameters, which were used to determine the IOM guidelines, cannot distinguish between fetal fat-mass (driven by GWG and the intrauterine environment) and fat-free mass that is genetically determined [26]. Additionally, the robust association of inadequate GWG with SGA status seen with both BMI classifications is also of public health importance and supports policies towards prevention of inadequate GWG, especially since SGA is also well-known to be associated with neonatal mortality and long-term cardiometabolic risk [28].

There is a paucity of South-East Asian data on the risk gradient for GWG and materno-foetal outcomes, with only a handful of studies from Thailand, Singapore, Indonesia, and Vietnam [13, 15, 16, 29, 30]. Our present study is only the second well-powered (n = 875) prospective analysis of a Malay-majority population, the first in recent-times and the only one to evaluate maternal insulin resistance. Insulin resistance is an important modulator of neonatal fat mass in both overweight/obese mothers with either normal glucose tolerance, or GDM [31–33]. Our finding that a pragmatic clinical variable such as excessive GWG is predictive of greater neonatal adiposity, independent of HOMA2%S, highlights the important role of antenatal weight management. It is therefore imperative that we raise awareness of this highly modifiable risk factor for offspring dysmetabolism, and consequently implement structured lifestyle modification programs during the antenatal period aimed at targeting optimal GWG.

Another key finding is the unexpected positive predictive power of the original IOM guidelines utilizing Caucasian BMI cut-offs in our cohort despite presumed ethnic, dietary, and cultural differences between East and West. This is reflective of the well-documented shift towards consumption of a Western diet observed in many developing Asian countries with greater prosperity. Data has revealed a transition to a diet based on 1) refined energy-dense carbohydrate sources that are wheat-based rather than the conventional rice-staple, 2) increased sugar and 3) processed foods in Malaysia thus resulting in epidemic obesity rates [19]. Despite a surface similarity with East Asian cohorts in terms of rice as the traditional carbohydrate staple, these data on societal changes in terms of diet/maternal obesity in Malaysia contrast with the high prevalence of maternal underweight in East Asian populations and emphasize how different our Malaysian cohort is from East Asian populations [18, 19, 34]. It also underlines the non-monolithic nature Asians who as a significant proportion of the global population display considerable ethnic and cultural heterogeneity.

To our knowledge, there have been only 3 other published reports originating from Singapore and Indonesia with a similar study design to ours, that explored the impact of GWG in South-East Asian cohorts which included Malay women [13–15]. Conflicting data on the applicability of IOM recommendations arising from these reports, are likely due to limitations of study design (retrospective, inadequate power) as well as differences between cohorts with regards to socio-economic status, nutrition, culture, and healthcare systems. Our findings that Caucasian BMI cut-offs are predictive of pregnancy outcomes echo those of a study conducted amongst Singaporeans recruited in 2010–2014, a population with similar dietary/cultural habits to Malaysia’s [13]. This prospective analysis (n = 704) applied 2009 IOM recommendations using only Caucasian BMI cut-offs, finding that excessive GWG was associated with macrosomia (adjusted OR 2.27) while inadequate GWG was linked with reduced neonatal adiposity (SGA: adjusted OR 2.97). Notably, the afore-mentioned Singaporean cohort had a Chinese-majority demographic distribution of 51.9% Chinese and 26.7% Malay ethnicity, whereas our Malaysian cohort had a composition of ~67% Malay and ~23% Chinese ethnicity. A retrospective analysis of a smaller Malaysian cohort of mothers (n = 436) (80% Malay ethnicity), also confirmed that low-GWG classified using Caucasian BMI cut-offs was associated with premature birth/low birth weight upon univariate analysis, but only in the normal BMI group [14]. This smaller Malaysian study conducted in 2010, however, was not sufficiently powered to determine increased neonatal adiposity outcomes.

In contrast to our findings, a large prospective study from Sumatra, Indonesia (n = 607) conducted in 2010, with a mono-ethnic Malay cohort, failed to find a significant association between excessive GWG and increased neonatal adiposity, with either Asian or Caucasian BMI cut-offs, but observed a correlation between inadequate GWG and SGA/prematurity with both Asian and Caucasian BMI cut-offs [15]. The absence of a significant correlation between excessive GWG and increased neonatal adiposity in this afore-mentioned study may be attributed to the low rates of excessive GWG, a high prevalence of underweight (20.1%) and inadequate GWG (~50% in those with low BMI and ~60% in normal BMI mothers), in contrast to our present Malaysian cohort and the Singaporean cohort [13, 15]. This could be secondary to the differing socio-economic status of these Sumatran mothers, only 46.5% of whom attained senior high school level education and 13.5% tertiary education. Indeed, the authors of the Indonesian study concluded that malnutrition might have contributed to the high prevalence of inadequate GWG in their antenatal cohort. In contrast to the results of the Indonesian study, observations from Thailand, another South-East Asian country albeit with a different non-Malay ethnic composition and socio-cultural practices, indicate that excessive GWG defined using Western BMI cut-off were well correlated with increased neonatal adiposity [16].

Broadly speaking, 3 approaches to managing GWG have been trialled in non-White populations: 1. Application of the original 2009 IOM guidelines with Caucasian BMI cut-offs 2. Modification of the 2009 IOM guidelines with country-specific BMI cut-offs whilst retaining IOM GWG reference ranges and 3. Development of national guidelines with country-specific BMI cut-offs AND customized GWG ranges for the local population. There is a lack of consensus and conflicting data as to which of these methods should guide antenatal care in Asian women. Overall, the published literature demonstrates that abnormal GWG categorized using Caucasian BMI cut-offs, as per the original 2009 IOM guidelines, correlates with adverse pregnancy outcomes in Asians. This is exemplified by corroborating data derived from East Asian and Singaporean cohorts and meta-analysis data [2, 13, 34]. There is also evidence that minor modifications of the 2009 IOM guidelines by transposing country-specific/regional BMI cut-offs on to the same GWG reference-ranges can predict adverse materno-fetal outcomes as borne out by both the landmark meta-analysis by Goldstein et al. and Asian studies from China, Japan and Korea [1, 3, 6, 7, 34]. The 3^rd^ approach of utilizing both local BMI cut-offs and GWG ranges has also been extensively validated in Japan [34]. Importantly, the majority of published Asian data are derived from ethnically homogeneous, East Asian cohorts which are exceptionally lean [2, 3, 6, 7, 34]. By way of example, the prevalence of underweight in Japan is reported to be 25%, whereas in our Malaysian cohort and the afore-mentioned Singaporean cohort only 7% and 8.4% respectively, were underweight [13, 35].

Recognizing the lack of Asian data, Goldstein et al., in a large meta-analysis across North America, Europe and Asia (including 8 Asian cohorts ie. China, Korea, Japan, Taiwan) found that use of 2009 IOM guidelines with Caucasian BMI cut-offs was strongly associated with pregnancy outcomes including SGA, LGA and macrosomia, in a pooled cohort of 1.3 million women [2]. When the same researchers conducted a subgroup analysis of the East Asian subjects (n = 318,143), they found that application of both regional Asian and Caucasian BMI cut-offs demonstrated elevated risk of adverse neonatal adiposity outcomes with inappropriate GWG [3]. These regional Asian BMI classifications were more commensurate with the Asian metabolic phenotype with maximum cut-offs of normal BMI as low as 23–24 kg/m^2^, whilst defining obesity as BMI ≥ 25 kg/m^2^ in Korean women and BMI ≥ 28 kg/m^2^ in Chinese mothers. Echoing our findings, the meta-analysis also found that application of regional Asian BMI cut-offs increased the proportion of women with excess GWG while lowering that with inadequate GWG, thus rendering the distribution of GWG categories more similar to that observed with Caucasian cut-offs [3]. Although these data indicate that the 2009 IOM recommendations are valid when applied using both Caucasian and regional Asian BMI cut-offs, the authors nevertheless recommended use of regional BMI cut-offs in Asians, as this mitigates risk of overestimating prevalence of inadequate GWG that is not clinically significant. This approach would also avoid underestimating incident excessive GWG. An important caveat to consider when interpreting the conclusions of this meta-analysis would be that the 8 Asian studies included were conducted in mono-ethnic East Asian samples and only 2 of the 8 were prospective analyses. As such, their results might not be applicable to our diverse Malaysian cohort. Our study, therefore, contributes valuable regional data of significant public health import. Mirroring the results of the aforementioned meta-analysis, we have demonstrated that using the 2009 IOM GWG recommendations with Caucasian cut-offs is also applicable to Malaysian women of Malay, Chinese and Indian descent. We have also shown that even after transposing Asian pre-pregnancy BMI cut-offs on to these IOM GWG recommendations, inappropriate GWG remains associated with an increased risk of abnormal neonatal anthropometrics, as borne out by Goldstein et al’s Asian subgroup analysis.

Ever more contemporaneous evidence is emerging that simple modification of IOM guidelines by transposing Asian BMI cut-offs on to existing IOM recommended weight-gain ranges (the 2^nd^ approach) are appropriate and preferred in the vast majority of Asian populations, including China. 2 more recent retrospective analyses from mainland China (Guan 2019) and Korea (Wie 2017) not included in the meta-analysis by Goldstein et al., found that these modified 2009 IOM guidelines are predictive of neonatal outcomes [3, 6, 7]. The mainland China study (n = 1593) used BMI >24 kg/m^2^ to define overweight and BMI >28 kg/m^2^ to define obesity as per Chinese Nutritional Society guidelines (approximating our Malaysian BMI cut-offs), whilst the Korean report (n = 7843) defined overweight as BMI 23–24.9 kg/m^2^ and obesity as ≥25 kg/m^2^ [6, 7].

A recent large comparative analysis from China contrasting application of the original 2009 IOM guidelines with national Chinese Nutritional Society (CNS) guidelines deploying Asian BMI cut-offs but the same GWG ranges as IOM, recommended that the Chinese adaptation should be preferred, as the model using Asian cut-offs had a higher sensitivity, specificity, positive predictive value, and negative predictive value for predicting offspring nutritional status in mothers with appropriate GWG. Based on these observations, the authors therefore concluded that the CNS guidelines were more suitable for use in Chinese women [1]. Further corroboration that the IOM GWG reference ranges are appropriate for use in Asians comes from a secondary analysis of prospectively collected data from the HAPO cohort in Hong Kong [26]. This report found that unique GWG reference-ranges derived from the Hong Kong cohort (with binary regression models correlating neonatal fat mass and maternal GWG) were fairly similar to that used by 2009 IOM guidelines. It was determined that optimal GWG ranges for Chinese women using Chinese BMI cut-offs previously alluded to were: 14.0–18.5 kg, 9.0–16.5 kg and 5.0–11.0 kg for under-, normal- and over-weight Chinese women, respectively.

The range of approaches laid out is mirrored by the substantial variability in the use/implementation of GWG guidance globally, as reported by recent reviews, with some countries adhering to the original 2009 IOM guidelines (North America/Finland/Australia), others modifying the IOM guidelines with regional BMI cut-offs (China), to countries like Vietnam/Japan utilizing completely bespoke local recommendations [36, 37]. There exists a wide spectrum of practices, ranging from countries with no GWG guidelines at all, to those that did not stratify GWG by maternal BMI, right through to New Zealand where ethnicity-specific BMI cut-offs are utilized for mothers of European-, Asian- and Pacific-descent [37, 38]. Consequent to the parlous lack of consensus and conflicting data, there have even been attempts to formulate an alternative to the 2009 IOM recommendations—the Intergrowth 21^st^ reference (including data from India and China), which was shown to have low sensitivity (despite high specificity) for adverse outcomes [39]. Given the paucity of existing Asian data, lack of standardization and uncertainty as to which guidelines should be applied, our study validating the use of the 2009 IOM GWG guidelines, adds to the existing literature, shedding light on a much under-studied but populous part of the world i.e. South-East Asia. As recommended by other experts, it is our belief that there is a pressing need for each country to formulate ethnicity-specific evidence-based national guidelines for its own population [8].

To our knowledge, this is one of a handful of South-East Asian studies in modern times to explore the relationship between pregnancy outcomes and inappropriate GWG stratified by both Asian and Caucasian BMI cut-offs. The use of a contemporary cohort accounts for prevailing increased rates of obesity after economic/nutritional transitions experienced by many Asian countries in recent times. Another strength was the prospective study design, with risk of adverse outcomes comprehensively adjusted for potential confounders including baby gender and insulin-resistance (given the propensity of Asians to exhibit higher insulin resistance at a lower BMI). It is also one of few studies to evaluate a Malay-majority population, a much under-studied ethnicity. Unlike some studies which correlated early pregnancy BMI with outcomes, we evaluated pre-pregnancy BMI–the basis and intention of the original IOM guidelines. Lastly, apart from birthweight, a conventional but crude indicator of adiposity, we also collected data on more sensitive discriminatory measures such as subcutaneous fat (ie. SSFT) which correlate better with future childhood risk [26].

Our study has several limitations. This was a single-centre study in a multi-ethnic population thus limiting generalizability of the results to other cohorts with differing demographics, socioeconomic status, cultural practices, and healthcare systems. Subjects were recruited based on convenience sampling. We did not fully account for the impact of maternal socio-economic status on neonatal anthropometrics however upon adjusting for educational level as a proxy for this there were no resultant major differences in outcomes (S5 Table). Data on maternal diet and physical activity were not collected. The sample size was not sufficiently large to derive meaningful sensitivity-analyses for GWG thresholds in each BMI category or detect differences between ethnic subgroups. The low event rate for LGA/BW >90^th^ centile is also a limitation of our study. Pre-pregnancy weight was based on maternal recall and subject to recollection bias. However, in order to mitigate this, the subject’s memory was prompted with the earliest available measured weight during pregnancy available in medical records at the time of booking in antenatal clinic. We did not assess other neonatal outcomes such as NICU admission and premature delivery. Foetal growth was assessed with Fenton 2013 growth charts (as Malaysia lacks homegrown charts), and this might have under-estimated LGA incidence. However, we circumvented this limitation by deriving customized cohort parameters such as BW/NFM/SSFT >90^th^ centile. Nevertheless, the use of standardized Fenton growth-centiles also facilitates comparison with other similar studies globally.

## Conclusion

Our prospective analysis confirms that abnormal GWG, as defined by 2009 IOM guidelines using both Caucasian and Asian BMI cut-offs, is independently associated with foetal under- and over-nutrition in a contemporary Malay-majority cohort. Uniquely, this study adjusted for the mediating impact of maternal insulin-resistance on neonatal anthropometrics. These observations therefore validate both use of the original 2009 IOM guidelines, and its modification with regional BMI cut-offs, for antenatal weight management of Malaysian women. Our observations can be of use in countries like Singapore with a similar multi-ethnic population inclusive of the Malay ethnic subgroup, and Indonesia which is the nation with the largest Malay population globally. The umbrella term “Asian” encompasses a multitude of diverse ethnicities with considerable biopsychosocial heterogeneity. Given the profound impact of GWG on the metabolic life-course of mother/child dyads with potential trans-generational metabolic consequences; there is a need for similar prospective studies in every region of Asia to establish national guidelines. Additionally periodic re-evaluation at 10-year intervals is required to capture the impact of interim socio-cultural transitions.

## Supporting information

S1 Appendix Details of screening, diagnosis, and standard of care for GDM, neonatal measurements and calculations, biochemical analyses. (DOCX)

S1 Table Neonatal outcomes and LSCS rate stratified by GWG category according to 2009 IOM guidelines, modified with Asian BMI cut-offs. (DOCX)

S2 Table Neonatal outcomes and LSCS rate stratified by GWG category according to 2009 IOM guidelines, using Caucasian BMI cut-offs. (DOCX)

S3 Table Maternal biochemical characteristics for entire cohort. (DOCX)

S4 Table Biochemical and clinical characteristics of mothers with GDM. (DOCX)

S5 Table Multiple logistic regression analysis for associations of increased and reduced neonatal anthropometrics adjusted for maternal education level. (DOCX)
