# Supplementary material for: Are the 2009 Institute of Medicine gestational weight gain recommendations applicable in a contemporary South-East Asian pregnancy cohort? Results of a prospective analysis
Source: PLoS One. 2025 Jan 6;20(1):e0316837. doi: 10.1371/journal.pone.0316837 (PMC11703048; doi:10.1371/journal.pone.0316837)
Supplement: S1 Appendix — (DOCX) [file pone.0316837.s001.docx]

**Appendix S1**

**Details of screening, diagnosis, and standard of care for GDM**Our institution practices universal screening with a 2-point 2-hour 75g oral glucose tolerance test (OGTT) as per Malaysian national guidelines. Gestational diabetes mellitus (GDM) is diagnosed if the woman has at least one abnormal plasma glucose reading (fasting plasma glucose ≥ 5.1mmol/L and/or 2-hour plasma glucose ≥ 7.8mmol/L) based on Malaysian diagnostic criteria.[1] Women with risk factors are screened at booking/first antenatal contact while all other women are screened at 24-28 weeks gestation.[1]

Once diagnosed, women with GDM are educated on medical nutrition therapy and asked to perform 7-point self-monitoring of blood glucose (SMBG) at least 3 days a week as per the usual practice standards in our institution.

The glycaemic targets for GDM in our institution are fasting 3.5 – 5.1mmol/L, pre-meals 4.0 – 5.8mmol/L, and 2-hours post-prandial 4.0 - 6.7mmol/l. If >30% of the SMBG values are beyond target despite compliance to medical nutrition therapy, insulin therapy is initiated.

Women with GDM are reviewed weekly if glucose levels are off-target and 2-weekly if within target, until 32-weeks gestation. After 36-weeks, all women are reviewed weekly until delivery.

**Neonatal measurements and calculations**

Baby weight was measured on a calibrated scale and length with a measuring board.

Skinfold thickness was measured with calibrated Harpenden calipers at 3 anatomical sites [2]:

1. Triceps: A vertical fold midway between the acromion process and the olecranon process (elbow).
2. Subscapular: A diagonal fold just below the inferior angle of the scapula.
3. Supra-iliac or flank: A diagonal fold just above the front forward protrusion of the hip bone (just above the iliac crest at the midaxillary line).

Neonatal fat mass (NFM):

Calculated based on the formula derived from Catalano et al. [3] i.e. 0·39055 (birth weight) + 0·0453 (flank skinfold) – 0·03237 (length) + 0·54657.

This method has been published previously and correlates strongly (r^2^ = 0·84) with estimates using total body electrical conductivity.

Sum of skinfold thickness (SSFT):

Flank skinfold + triceps skinfold + subscapular skinfold (mm)

This method has been used by the “Hyperglycaemia and Adverse Pregnancy Outcome” (HAPO) Study Cooperative Research Group in their study for evaluating the association of hyperglycaemia in pregnancy with neonatal anthropometrics.[4]

**Biochemical analyses**

Blood samples for insulin were centrifuged at 4°C and the plasma separated and stored at -80°C until subsequent analysis. Plasma glucose was measured with an automatic analyzer (Yellow Springs Instruments, Ohio) using a glucose oxidase method. Plasma insulin was measured by radioimmunoassay.

**References**

1. Clinical Practice Guidelines: Management of Type 2 Diabetes Mellitus. 5 ed. Putrajaya: Malaysia Health Technology Assessment Section (MaHTAS), Ministry of Health Malaysia; 2015.

2. Simple measures - skinfolds: MRC Epidemiology Unit; [cited 2024 July 27]. Available from: <https://www.measurement-toolkit.org/anthropometry/objective-methods/simple-measures-skinfolds>.

3. Catalano PM, Thomas AJ, Avallone DA, Amini SB. Anthropometric estimation of neonatal body composition. Am J Obstet Gynecol. 1995;173(4):1176-81. doi: 10.1016/0002-9378(95)91348-3. PubMed PMID: 7485315.

4. Group HSCR. Hyperglycemia and Adverse Pregnancy Outcome (HAPO) Study: associations with neonatal anthropometrics. Diabetes. 2009;58(2):453-9. Epub 20081114. doi: 10.2337/db08-1112. PubMed PMID: 19011170; PubMed Central PMCID: PMCPMC2628620.
