# Supplementary material for: Are the 2009 Institute of Medicine gestational weight gain recommendations applicable in a contemporary South-East Asian pregnancy cohort? Results of a prospective analysis
Source: PLoS One. 2025 Jan 6;20(1):e0316837. doi: 10.1371/journal.pone.0316837 (PMC11703048; doi:10.1371/journal.pone.0316837)
Supplement: S1 Table — (DOCX) [file pone.0316837.s002.docx]

**Table S1: Neonatal outcomes and LSCS rate stratified by GWG category according to 2009 IOM guidelines, modified with Asian BMI cut-offs**

| Neonatal outcomes | GWG according to IOM recommendation (Asian BMI cut-offs) | | | | | | p-value |
| --- | --- | --- | --- | --- | --- | --- | --- |
|  | N | Inadequate | N | Adequate | N | Excessive |  |
| Gestation at delivery (weeks)^**^ | 218 | 38·20 ± 1·09 | 302 | 38·25 ± 1·08 | 355 | 38·43 ± 1·14 | 0·023 |
| Birthweight (g), Mean ± SD^*/**/***^ | 218 | 2923·52 ± 344·12 | 302 | 3077·88 ± 364·88 | 355 | 3180·44 ± 379·40 | <0·001 |
| Fat mass (g), ^†^ Mean ± SD^*/**^ | 120 | 368·32 ± 116·42 | 137 | 442·18 ± 139·08 | 164 | 475·62 ± 149·34 | <0·001 |
| Sum of skinfold thickness (mm), ^†^  Mean ± SD^*/**/***^ | 119 | 14·58 ± 3·11 | 137 | 15·97 ± 3·14 | 162 | 17·21 ± 3·95 | <0·001 |
|  |  |  |  |  |  |  |  |
| Macrosomia, N (%) ^*/**/***^ | 218 |  | 302 |  | 355 |  | <0·001 |
| BW <2·5kg |  | 24 (11·0) |  | 16 (5·3) |  | 8 (2·3) |  |
| BW 2·5 – 3·9kg |  | 194 (89·0) |  | 285 (94·4) |  | 337 (94·9) |  |
| BW ≥4kg |  | 0 (0·0) |  | 1 (0·3) |  | 10 (2·8) |  |
|  |  |  |  |  |  |  |  |
| Baby size, N (%) ^*/**^ | 218 |  | 302 |  | 355 |  | <0·001 |
| SGA |  | 50 (22·9) |  | 26 (8·6) |  | 22 (6·2) |  |
| AGA |  | 167 (76·6) |  | 271 (89·7) |  | 320 (90·1) |  |
| LGA |  | 1 (0·5) |  | 5 (1·7) |  | 13 (3·7) |  |
|  |  |  |  |  |  |  |  |
| Birth weight, N (%) ^*/**/***^ | 218 |  | 302 |  | 355 |  | <0·001 |
| BW <10^th^ centile (<2628g) |  | 36 (16·5) |  | 31 (10·3) |  | 20 (5·6) |  |
| BW 10 – 90^th^ centile (2628 -3590g) |  | 172 (78·9) |  | 242 (80·1) |  | 288 (81·1) |  |
| BW >90^th^ centile (>3590g) |  | 10 (4·6) |  | 29 (9·6) |  | 47 (13·2) |  |
|  |  |  |  |  |  |  |  |
| Fat mass (g), N (%) ^*/**^ | 120 |  | 137 |  | 164 |  | <0·001 |
| NFM <10^th^ centile (<264·3g) |  | 24 (20·0) |  | 10 (7·3) |  | 8 (4·9) |  |
| NFM 10 – 90^th^ centile (264·3g – 617·2g) |  | 95 (79·2) |  | 114 (83·2) |  | 128 (78·0) |  |
| NFM >90^th^ centile (>617·2g) |  | 1 (0·8) |  | 13 (9·5) |  | 28 (17·1) |  |
|  |  |  |  |  |  |  |  |
| Sum of skinfold thickness (mm), N (%) ^*/**/***^ | 119 |  | 137 |  | 162 |  | <0·001 |
| SSFT <10^th^ centile (<11·5mm) |  | 21 (17·7) |  | 8 (5·8) |  | 11 (6·8) |  |
| SSFT 10 – 90^th^ centile (11·5 – 21·0mm) |  | 97 (81·5) |  | 122 (89·1) |  | 119 (73·4) |  |
| SSFT >90^th^ centile (>21·0mm) |  | 1 (0·8) |  | 7 (5·1) |  | 32 (19·8) |  |
|  |  |  |  |  |  |  |  |
| LSCS, N (%) ^**^ | 218 |  | 302 |  | 355 |  | 0·121 |
| Yes |  | 64 (29·4) |  | 103 (34·1) |  | 134 (37·7) |  |
| No |  | 154 (70·6) |  | 199 (65·9) |  | 221 (62·3) |  |

BMI, body mass index; GWG, gestational weight gain; BW, birth weight; SGA, small for gestational age; AGA, appropriate for gestational age; LGA, large for gestational age; NFM, neonatal fat mass; SSFT, sum of skinfold thickness; LSCS, lower segment Caesarean section.

The 10^th^ and 90^th^ centile of birth weight, neonatal fat mass, neonatal sum of skinfold thickness for this cohort were determined using SPSS. Continuous data expressed as means ± SDs and analysed using one-way analysis of variance (ANOVA) and Tukey’s post hoc analysis. † Welch ANOVA used due to unequal variance in Levene’s test. Categorical data are expressed as percentages and analysed using the chi-square or Fisher’s exact test. Post hoc analysis: *P < 0·05, Inadequate vs Adequate; **P < 0·05, Inadequate vs Excessive; ***P < 0·05, Adequate vs Excessive.
