# Supplementary material for: Are the 2009 Institute of Medicine gestational weight gain recommendations applicable in a contemporary South-East Asian pregnancy cohort? Results of a prospective analysis
Source: PLoS One. 2025 Jan 6;20(1):e0316837. doi: 10.1371/journal.pone.0316837 (PMC11703048; doi:10.1371/journal.pone.0316837)
Supplement: S2 Table — (DOCX) [file pone.0316837.s003.docx]

**Table S2: Neonatal outcomes and LSCS rate stratified by GWG category according to 2009 IOM guidelines, using Caucasian BMI cut-offs**

| Neonatal outcomes | GWG according to IOM recommendation (Caucasian BMI) | | | | | | p-value |
| --- | --- | --- | --- | --- | --- | --- | --- |
|  | N | Inadequate | N | Adequate | N | Excessive |  |
| Gestation at delivery (weeks)^**^ | 263 | 38·19 ± 1·07 | 314 | 38·28 ± 1·13 | 298 | 38·46 ± 1·11 | 0·013 |
| Birthweight (g), Mean ± SD^*/**/***^ | 263 | 2938·43 ± 348·30 | 314 | 3088·24 ± 361·46 | 298 | 3199·30 ± 382·43 | <0·001 |
| Fat mass (g), ^†^ Mean ± SD^*/**/***^ | 140 | 376·19 ± 117·11 | 147 | 442·28 ± 139·90 | 134 | 485·80 ± 152·39 | <0·001 |
| Sum of skinfold thickness (mm), ^†^  Mean ± SD^*/**/***^ | 139 | 14·78 ± 3·04 | 146 | 16·00 ± 3·45 | 133 | 17·45 ± 3·88 | <0·001 |
|  |  |  |  |  |  |  |  |
| Macrosomia, N (%) ^*/**/***^ | 263 |  | 314 |  | 298 |  | <0·001 |
| BW <2·5kg |  | 27 (10·3) |  | 16 (5·1) |  | 5 (1·7) |  |
| BW 2·5 – 3·9kg |  | 236 (89·7) |  | 297 (94·6) |  | 283 (95·0) |  |
| BW ≥4kg |  | 0 (0·0) |  | 1 (0·3) |  | 10 (3·3) |  |
|  |  |  |  |  |  |  |  |
| Baby size, N (%) ^*/**^ | 263 |  | 314 |  | 298 |  | <0·001 |
| SGA |  | 58 (22·1) |  | 22 (7·0) |  | 18 (6·0) |  |
| AGA |  | 204 (77·5) |  | 287 (91·4) |  | 267 (89·6) |  |
| LGA |  | 1 (0·4) |  | 5 (1·6) |  | 13 (4·4) |  |
|  |  |  |  |  |  |  |  |
| Birth weight, N (%) ^*/**/***^ | 263 |  | 314 |  | 298 |  | <0·001 |
| BW <10^th^ centile (<2628g) |  | 44 (16·7) |  | 28 (8·9) |  | 15 (5·0) |  |
| BW 10 – 90^th^ centile (2628 -3590g) |  | 207 (78·7) |  | 256 (81·5) |  | 239 (80·2) |  |
| BW >90^th^ centile (>3590g) |  | 12 (4·6) |  | 30 (9·6) |  | 44 (14·8) |  |
|  |  |  |  |  |  |  |  |
| Fat mass (g), N (%) ^*/**/***^ | 140 |  | 147 |  | 134 |  | <0·001 |
| NFM <10^th^ centile (<264·3g) |  | 25 (17·9) |  | 12 (8·2) |  | 5 (3·7) |  |
| NFM 10 – 90^th^ centile (264·3g – 617·2g) |  | 113 (80·7) |  | 121 (82·3) |  | 103 (76·9) |  |
| NFM >90^th^ centile (>617·2g) |  | 2 (1·4) |  | 14 (9·5) |  | 26 (19·4) |  |
|  |  |  |  |  |  |  |  |
| Sum of skinfold thickness (mm), N (%) ^*/**/***^ | 139 |  | 146 |  | 133 |  | <0·001 |
| SSFT <10^th^ centile (<11·5mm) |  | 20 (14·4) |  | 13 (8·9) |  | 7 (5·3) |  |
| SSFT 10 – 90^th^ centile (11·5 – 21·0mm) |  | 117 (84·2) |  | 122 (83·6) |  | 99 (74·4) |  |
| SSFT > 90^th^ centile (>21·0mm) |  | 2 (1·4) |  | 11 (7·5) |  | 27 (20·3) |  |
|  |  |  |  |  |  |  |  |
| LSCS, N (%) | 263 |  | 314 |  | 298 |  | 0·171 |
| Yes |  | 81 (30·8) |  | 106 (33·8) |  | 114 (38·3) |  |
| No |  | 182 (69·2) |  | 208 (66·2) |  | 184 (61·7) |  |

BMI, body mass index; GWG, gestational weight gain; BW, birth weight; SGA, small for gestational age; AGA, appropriate for gestational age; LGA, large for gestational age; NFM, neonatal fat mass; SSFT, sum of skinfold thickness; LSCS, lower segment caesarean section.

The 10^th^ and 90^th^ centile of birth weight, neonatal fat mass, neonatal sum of skinfold thickness for this cohort were determined using SPSS. Continuous data expressed as means ± SDs and analysed using one-way analysis of variance (ANOVA). † Welch ANOVA used due to unequal variance in Levene’s test and Tukey’s post hoc analysis. Categorical data are expressed as percentages and analysed using the chi-square or Fisher’s exact test. Post hoc analysis: *P < 0·05, Insufficient vs Adequate; **P < 0·05, Insufficient vs Excessive; ***P < 0·05, Adequate vs Excessive.
