# Supplementary material for: Are the 2009 Institute of Medicine gestational weight gain recommendations applicable in a contemporary South-East Asian pregnancy cohort? Results of a prospective analysis
Source: PLoS One. 2025 Jan 6;20(1):e0316837. doi: 10.1371/journal.pone.0316837 (PMC11703048; doi:10.1371/journal.pone.0316837)
Supplement: S3 Table — (DOCX) [file pone.0316837.s004.docx]

**Table S3: Maternal biochemical characteristics for entire cohort**

| Biochemical characteristics at recruitment | N | Mean ± SD / Median (IQR) |
| --- | --- | --- |
| Fasting glucose, OGTT (mmol/L) | 873 | 4.4 ± 0.5 |
| 2-hour glucose, OGTT (mmol/L) | 873 | 6.6 ± 1.6 |
| AUC Glucose (mmol_h/L) | 873 | 10.9 ± 1.8 |
| Fasting Insulin (mIU/L), | 858 | 11.7 (8.3 – 16.6) |
| Fasting C-peptide (ng/mL) | 853 | 1.7 (1.2 – 2.4) |
| AUC Insulin (mIU_h/L) | 709 | 100.7 (63.9 – 167.9) |
| AUC insulin to AUC glucose ratio | 709 | 10.0 (6.5 – 15.0) |
| HOMA-2%S | 852 | 69.7 (49.2 – 98.9) |
| HOMA-2%B | 853 | 149.7 (122.2 – 181.7) |
| Fasting Tg, (mmol/L) | 857 | 2.0 (1.6 – 2.6) |
|  |  |  |
| Biochemical characteristics at 36 weeks of gestation | N | Mean ± SD / Median (IQR) |
| Fasting glucose (mmol/L) | 437 | 4.3 ± 0.5 |
| HbA1c (%) | 463 | 5.3 ± 0.4 |
| Fasting Insulin (mIU/L) | 429 | 13.0 (9.5 – 18.2) |
| Fasting C-peptide (ng/mL) | 426 | 2.0 (1.5 – 2.8) |
| HOMA-2%S | 427 | 62.7 (44.4 – 86.3) |
| HOMA-2%B | 426 | 177.0 (142.8 – 214.8) |
| Fasting Tg, (mmol/L) | 426 | 2.8 (2.2 – 3.4) |

OGTT, oral glucose tolerance test; AUC, area under the curve; Tg, triglyceride; HbA1c, glycosylated haemoglobin; HOMA2-%B, updated homeostasis model for assessment of β-cell function; HOMA2-%S, updated homeostasis model for assessment of insulin sensitivity. All data presented as mean ± SD or median (IQR).
