# Supplementary material for: Are the 2009 Institute of Medicine gestational weight gain recommendations applicable in a contemporary South-East Asian pregnancy cohort? Results of a prospective analysis
Source: PLoS One. 2025 Jan 6;20(1):e0316837. doi: 10.1371/journal.pone.0316837 (PMC11703048; doi:10.1371/journal.pone.0316837)
Supplement: S4 Table — (DOCX) [file pone.0316837.s005.docx]

**Table S4: Biochemical and clinical characteristics of mothers with GDM**

| Biochemical characteristics at recruitment | N | Mean ± SD / Median (IQR) |
| --- | --- | --- |
| Fasting glucose, OGTT (mmol/L) | 260 | 4·8 ± 0·7 |
| 2-hour glucose, OGTT (mmol/L) | 260 | 8·1 ± 1·5 |
| AUC Glucose (mmol_h/L) | 260 | 12·9 ± 1·5 |
| Fasting Insulin (mIU/L), | 250 | 12·9 (8·6 – 19·9) |
| Fasting C-peptide (ng/mL) | 250 | 2·0 (1·2 – 2·9) |
| AUC Insulin (mIU_h/L) | 125 | 163·8 (108·5 – 251·4) |
| AUC insulin to AUC glucose ratio | 125 | 13·2 (8·5 – 19·2) |
| HOMA-2%S | 248 | 61·0 (41·0 – 94·9) |
| HOMA-2%B | 248 | 142·0 (112·3 – 182·0) |
| Fasting Tg, (mmol/L) | 249 | 2·2 (1·8 – 2·8) |
|  |  |  |
| Biochemical characteristics at 36 weeks of gestation | **N** | **Mean ± SD / Median (IQR)** |
| Fasting glucose (mmol/L) | 148 | 4·4 ± 0·7 |
| HbA1c (%) | 173 | 5·4 ± 0·5 |
| Fasting Insulin (mIU/L) | 139 | 13·0 (9·3 – 18·7) |
| Fasting C-peptide (ng/mL) | 138 | 2·0 (1·4 – 2·8) |
| HOMA2-%S | 138 | 62·0 (43·4 – 89·1) |
| HOMA2-%B | 138 | 165·7 (141·3 – 206·5) |
| Fasting Tg, (mmol/L) | 138 | 3·0 (2·5 – 3·5) |

OGTT, oral glucose tolerance test; AUC, area under the curve; Tg, triglyceride; HbA1c, glycosylated haemoglobin; HOMA2-%B, updated homeostasis model for assessment of β-cell function; HOMA2-%S, updated homeostasis model for assessment of insulin sensitivity. All data presented as mean ± SD or median (IQR).
