# Supplementary material for: Are the 2009 Institute of Medicine gestational weight gain recommendations applicable in a contemporary South-East Asian pregnancy cohort? Results of a prospective analysis
Source: PLoS One. 2025 Jan 6;20(1):e0316837. doi: 10.1371/journal.pone.0316837 (PMC11703048; doi:10.1371/journal.pone.0316837)
Supplement: S5 Table — (DOCX) [file pone.0316837.s006.docx]

**Table S5: Multiple logistic regression analysis for associations of increased and reduced neonatal anthropometrics adjusted for maternal education level**

| Neonatal outcomes | Adjusted OR (95% CI) | | | |
| --- | --- | --- | --- | --- |
|  | Inadequate GWG  (Asian BMI cut-offs)* | Excessive GWG  (Asian BMI cut-offs)* | Inadequate GWG  (Caucasian BMI cut-offs)* | Excessive GWG  (Caucasian BMI cut-offs)* |
| BW <10^th^ centile^¥^ | 1.91 (1.09 – 3.37) | 0.52 (0.28 – 0.98) | 2.32 (1.33 – 4.04) | 0.54 (0.27 – 1.08) |
| SGA | 3.63 (2.11 – 6.25) | 0.69 (0.37 – 1.29) | 4.26 (2.46 – 7.38) | 0.78 (0.40 – 1.55) |
| BW <2.5kg | 2.24 (1.12 – 4.48) | 0.39 (0.16 – 0.95) | 2.04 (1.04 – 4.00) | 0.28 (0.10 – 0.79) |
| NFM <10^th^ centile^¥^ | 4.14 (1.76 – 9.71) | 0.49 (0.18 – 1.36) | 2.97 (1.33 – 6.60) | 0.33 (0.10 – 1.02) |
| SSFT <10^th^ centile^¥^ | 1.99 (0.81 – 4.89) | 0.90 (0.33 – 2.43) | 1.32 (0.57 – 3.02) | 0.60 (0.21 – 1.70) |
| BW >90^th^ centile^¥^ | 0.49 (0.23 – 1.06) | 1.17 (0.68 – 1.99) | 0.47 (0.23 – 0.98) | 1.54 (0.89 – 2.66) |
| LGA | 0.25 (0.03 – 2.30) | 2.01 (0.68 – 5.92) | 0.20 (0.02 – 1.82) | 2.61 (0.88 – 7.70) |
| Macrosomia | € | 6.08 (0.75 – 49.43) | € | 8.54 (1.05 – 69.59) |
| NFM >90^th^ centile^¥^ | 0.07 (0.01 – 0.63) | 1.73 (0.82 – 3.68) | 0.12 (0.03 – 0.59) | 2.06 (0.98 – 4.34) |
| SSFT>90^th^ centile^¥^ | 0.16 (0.02 – 1.34) | 5.86 (2.39 – 14.41) | 0.17 (0.04 – 0.79) | 3.94 (1.79 – 8.69) |

*Adjusted for age, parity, race, GDM, Ln HOMA2%S and baby gender, ¥Adjusted for gestational age at delivery. € no event in the inadequate weight gain group. The 10th centile of BW, NFM and SSFT were 2628g, 264.26g and 11.5mm respectively (determined using SPSS).
